# Intergenerational Effects of Sublethal Lambda-Cyhalothrin Exposure on *Aphis gossypii* Glover (Hemiptera: Aphididae) Reproduction and Development

**DOI:** 10.3390/insects15030173

**Published:** 2024-03-04

**Authors:** Yuepeng Qiu, Zhaorong Chen

**Affiliations:** College of Horticulture and Landscape, Tianjin Agricultural University, Tianjin 300392, China

**Keywords:** pyrethroid pesticides, toxicity, life tables, agricultural pest control, resistance management

## Abstract

**Simple Summary:**

*Aphis gossypii* Glover is a pervasive pest with a global presence, causing significant economic losses in agriculture. Lambda-cyhalothrin is a widely used pyrethroid insecticide. Former research has shown that numerous chemical insecticides have a sublethal effect on *A. gossypii*. However, the sublethal effect of lambda-cyhalothrin on *A. gossypii* remains unknown. This study examined the effects of a sublethal dose of lambda-cyhalothrin on *A. gossypii* by life tables and qPCR analysis. In conclusion, the results clarified the sublethal effect of lambda-cyhalothrin on *A. gossypii* and provided a theoretical foundation for the prudent utilization of insecticides to combat this pest and devise strategies for managing resistance.

**Abstract:**

*Aphis gossypii* Glover, a widespread insect, presents a substantial danger to global agriculture. Lambda-cyhalothrin is a pyrethroid insecticide that has been widely studied for its effects on arthropods. Studies have reported that sublethal doses of insecticides can produce various consequences on arthropod reproduction. Hence, the objective of this research was to examine the potential effects of a sublethal dose of lambda-cyhalothrin (LC_30_, 1.15 mg/L) on *A. gossypii*, for which we created life tables and conducted qPCR analysis. Adult longevity, fecundity, net reproductive rate (*R*_0_), body length, width, weight, and the expression of vitellogenin (*Vg*) and vitellogenin receptor (*VgR*) genes were not significantly altered by lambda-cyhalothrin treatment at LC_30_ concentration in the F_0_ generation of *A. gossypii* adults. The intrinsic rate of increase (*r*) and finite rates of increase (*λ*) decreased significantly, while the mean generation time (*T*) increased. In addition, *Vg* and *VgR* gene expression levels were significantly higher in the F_1_ and F_2_ generations, whereas body length, width, and weight were notably reduced. The developmental duration, longevity, *r,* and *λ* did not differ significantly from those of the control group. Thus, the sublethal and intergenerational stimulatory effects of lambda-cyhalothrin were observed in *A. gossypii*, and the alterations in *Vg* and *VgR* in *A. gossypii* were strongly associated with sublethal effects. The results of this research offer valuable knowledge regarding the indirect impacts of lambda-cyhalothrin on *A. gossypii*, which can be utilized as a theoretical foundation for the prudent utilization of insecticides to combat this pest and devise strategies for managing resistance.

## 1. Introduction

*Aphis gossypii* Glover (Hemiptera: Aphididae), a pest with piercing–sucking mouthparts, is a global threat to a wide range of hosts and causes considerable economic losses in agriculture [1,2]. At present, the main approach for managing this pest is the use of chemical control. However, due to the widespread utilization of chemical insecticides, *A. gossypii* has acquired resistance to organophosphates [3], neonicotinoids [4], pyrethroids [5], and various other insecticides. Additionally, the sublethal doses of insecticides can stimulate *A. gossypii* populations over multiple generations, a phenomenon known as intergenerational effect. Specifically, it has an indirect effect on the progeny. For instance, exposing *A. gossypii* to the sublethal concentrations of nitenpyram resulted in cross-generational excitability in progenies [6]. Similarly, the treatment of *A. gossypii* with sulfoxaflor at LC_20_ led to significant increases in the fecundity of both F_1_ and F_2_ generations [7].

Pyrethroid pesticides are a type of insecticide that emerged after organophosphorus, organochlorine, and carbamate pesticides, and are characterized by their high activity and environmental compatibility. They are the major pesticide species in chemical control [8]. Studies conducted recently on the indirect and multigenerational impacts of pyrethroid insecticides in *A. gossypii* have revealed that LC_30_ decamethrin can reduce the intrinsic rate of increase in *A. gossypii* in the G_0_ generation. However, the intrinsic rates of increase in G_1_ and G_2_ were significantly higher [9]. Lambda-cyhalothrin is a new generation of pyrethroid pesticides developed by Imperial Chemical Industries (ICI) (now part of Syngenta) in the UK in 1984. It has the characteristics of low toxicity, high efficiency, and environmental protection. Its mechanism of action involves disrupting neural system function by interacting with pest sodium channels [10]. Consequently, it is widely used in the control of pests, such as *Pieris rapae*, Aphididae, *Helicoverpa armigera*, and *Spodoptera exigua*. In particular, its combination with neonicotinoid insecticides has a good effect [11]. However, with the long-term application, the resistance of pests continues to increase, and certain risks emerge for beneficial insects [12,13]. Studies have shown that *Phaedon cochleariae* reduced fecundity and body weight after exposure to lambda-cyhalothrin [14], and that the fecundity of F_0_ and F_1_ generations of *Lygus pratensis* and *Polymerus cognatus* was reduced by treatment with LD_50_ of lambda-cyhalothrin [15]. Additionally, fecundity was markedly reduced by lambda-cyhalothrin treatment in *Chrysoperla sinica* [16].

Vitellogenin (*Vg*) plays a crucial role in the reproductive process as an essential protein, providing necessary nutrients for embryonic growth and controlling vitellogenesis. This process directly impacts fecundity by regulating the development of the ovaries [17,18,19]. Vitellogenin receptors (*VgR*) are the basis of vitellogenesis and are critical for the maturation of insect ovaries [20]. Research has shown that the low doses of insecticides impact the manifestation of *Vg* and *VgR* genes in insects. While some insecticides inhibit the expression of *Vg* genes, thereby reducing fecundity, others promote reproduction by stimulating *Vg* expression [21,22]. For example, exposure to the sublethal concentrations of clothianidin (LC_5_ and LC_15_), chlorantraniliprole, and sulfoxaflor downregulated *Vg* expression levels in *A. gossypii* [23], *Chilo suppressalis* [24], and female adults of *Coccinella septempunctata* [25] and *Apolygus lucorum* [26], respectively, and ultimately affected the development and reproduction of their progenies. In addition, the expression of *Vg* and *VgR* in *Conopomorpha sinensis* adults [27] and *Sogatella furcifera* [28] was significantly inhibited by emamectin benzoate at LC_10_ and LC_30_, as well as thiamethoxam at LC_10_, resulting in a significant decrease in their reproductive capacity. In contrast, exposure to sublethal doses of sulfoxaflor (LC_10_), triazophos and decamethrin (LC_20_), and decamethrin and imidacloprid significantly upregulated *Vg* expression levels in *A. gossypii* [29], *Cyrtorhinus lividipennis* first-feathered adult females [30], and *Nilaparvata Lugens* adult females [31], respectively. Additionally, the LC_25_ of triazophos markedly stimulated the expression of *Vg*, *Vg-like*, and *VgR* in *Sogatella furcifera* [28]. To summarize, insecticides have the ability to control the production and activity of *Vg* genes, thus either promoting or hindering insect procreation. Hence, the effect of sublethal doses of various insecticides on insect *Vg* and *VgR* genes must be investigated in order to understand insect procreation-related sublethal consequences.

This study aimed to evaluate the effects of lambda-cyhalothrin on *A. gossypii*, and to examine its sublethal and intergenerational effects on *A. gossypii* through the creation of a life table. Additionally, to explore the effect of sublethal dose of lambda-cyhalothrin on the reproduction of *A. gossypii*, the expression levels of *Vg* and *VgR* gene were determined. The results of this research provide a theoretical foundation for the scientific application of insecticides in managing *A. gossypii* and devising efficient strategies for resistance management.

## 2. Materials and Methods

### 2.1. Experimental Materials

*A. gossypii* were procured from the Institute of Cotton Research of CAAS (Anyang, China) and reared for more than 10 generations on cotton leaves (CCRI 49) in a laboratory-controlled environment that maintained a constant temperature of 25 ± 1 °C and relative humidity of 65 ± 5%, with a 14 h light/10 h dark cycle, without any exposure to insecticides.

Lambda-cyhalothrin original drug (95%) was supplied by Hubei Marvel-Bio Medicine Co., Ltd. (Wuhan, China).

### 2.2. Experimental Method

#### 2.2.1. Toxicity of Lambda-Cyhalothrin to *A. gossypii*

The toxicity of lambda-cyhalothrin to *A. gossypii* was determined using the FAO-recommended aphid dip test [32]. For the pesticide treatment groups, lambda-cyhalothrin was dissolved in acetone and diluted with 0.01% Triton X-100 to create seven concentration gradients (128, 64, 32, 16, 8, 4, and 2 mg/L). The solution containing the same concentration of acetone and 0.01% Triton X-100 was used as a control. Healthy, wingless *A. gossypii* adults were selected and immersed in the drug liquid for 5 s before being removed and surface dried. The treated insects were then placed on fresh cotton leaves in 1.8% agar Petri dishes with 30 heads per concentration and four biological replicates. The Petri dishes were covered with paper towels underneath the lid to prevent insects from escaping. After 48 h, the number of deaths of the test insects was recorded and the mortality rate was calculated. Subsequently, all treated insects were placed in an incubator for feeding.

#### 2.2.2. Life Table Construction

Initially, adult wingless *A. gossypii* was placed in 1.8% agar Petri dishes containing fresh cotton leaves. The adult aphids were taken out after 24 h, and the newborn nymphs were collected and fed for 5 d to reach the adult stage as the F_0_ generation. According to the results of biological test, the treatment group was exposed to a solution of LC_30_ lambda-cyhalothrin, whereas the control group was exposed to 0.01% Triton X-100 containing the corresponding concentration of acetone. The F_0_ generation of *A. gossypii* adult (<24 h) was immersed in the treatment and control groups for 5 s each, respectively. After drying the drug liquid, the insects were transferred onto the fresh cotton leaves in 1.8% agar Petri dishes. They were fed one head on each leaf, with a paper towel underneath the lid of the Petri dish to prevent them from escaping. Subsequently, the growth status of the insects was monitored every 24 h, and the number of offspring and deaths were recorded. Newborn aphids produced in the F_0_ generation were used as the F_1_ generation, and those produced in the F_1_ generation were used as the F_2_ generation, which continued to be fed in a Petri dish with a single head. Their life table data were recorded until all test insects died. Ninety biological replicates were used for each treatment. While recording the life table data, the body length and body width of adult aphids of each generation were photographed and measured for 48 h using an electron microscope (Sz61, Olympus Corporation, Tokyo, Japan). The body weights of the aphids were measured using an analytical balance (XS205DU, Mettler Toledo, Zurich, Switzerland). At least 30 aphids were measured for each treatment group. All the *A. gossypii* used in the test were placed in an incubator. The experiment involved replacing the leaves and Petri dishes every 2–3 days.

#### 2.2.3. Sample Collection, Total RNA Extraction, and cDNA Library Construction

*A. gossypii* adults (48 h) in the F_0_–F_2_ generations treatment groups and the control group were collected separately in the RNase-free centrifuge tubes. Each treatment was replicated three times, and at least 50 *A. gossypii* adults were collected from each. The samples were cryogenically preserved in liquid nitrogen and kept in a freezer at −80 °C for the purpose of extracting RNA.

Total RNA from adult insects was extracted using TRIzol reagent (Invitrogen, Carlsbad, CA, USA), and the quality of the RNA was assessed using a spectrophotometer (Nanodrop2000c, Thermo Scientific, Waltham, MA, USA).

The cDNA synthesis was carried out using the MonScript^TM^ 5×RTⅢ All-in-One Mix kit (Monad Biotech Co., Ltd., Wuhan, China) according to the manufacturer’s instructions, with the extracted RNA (1 μL) serving as a template. The synthesized cDNA was diluted 20 times and stored at −20 °C.

#### 2.2.4. Quantitative Real-Time PCR

RT-qPCR was conducted on a StepOnePlus™ Real-Time PCR System (CFX Opus 96; Bio-Rad, Singapore). The qPCR reaction system was synthesized according to the instructions provided for MonAmp^TM^ SYBR^®^Green qPCR Mix (Monad Biotech Co., Ltd., Wuhan, China). The reactions included 10 μL qPCR Mix, 1 μL cDNA, 0.4 μL primer F/R, and Nuclease-Free Water added to 20 μL. The reaction program was set as follows: 95 °C for 30 s, 40 cycles of 95 °C for 10 s, and 60 °C for 30 s. Three mechanical and three technical replicates were used for each treatment. The *β-actin* of *A. gossypii* was used as an internal reference gene, and the primer sequences of genes *Vg* and *VgR* were designed as described by Ma et al. [33] and Wang et al. [34] (Table S1). The primers were synthesized by Sangon Biotech Co., Ltd. (Shanghai, China). The relative expression of *Vg* and *VgR* was calculated by Livak and Schmittgen [35]’s 2^−ΔΔCt^ method.

### 2.3. Data Analysis

To calculate the virulence regression formula, raw test data were analyzed using the PROBIT model [36] in IBM SPSS Statistics 20.0 (IBM, Chicago, IL, USA), from which the LC_30_ and 95% confidence interval for sublethal concentrations were calculated. Additionally, the significance of body length, width, and weight measurements, as well as the qPCR results of *A. gossypii*, were assessed through the utilization of analysis of variance (ANOVA) and Student’s *t*-test.

To calculate the developmental time of each stage, longevity, fecundity, net reproductive rate (*R*_0_), intrinsic rate of increase (*r*), finite rate of increase (*λ*), mean generation time (*T*), age-stage specific survival rate (*S_xj_*), age-specific survival rate (*l_x_*), age-specific fecundity (*m_x_*), age-specific maternity (*l_x_m_x_*), age-stage specific life expectancy (*e_xj_*), age-stage reproductive value (*v_xj_*), and other life table parameters of *A. gossypii*, raw life table data were processed using TWOSEX-MSChart (http://140.120.197.173/Ecology/, accessed on 12 August 2022) [37,38,39,40]. In addition, the bootstrap technique was used with 100,000 random resampling tests to determine the mean and SE of life table parameters, and the paired bootstrap method was used to analyze the significant differences between the treatment group and the control group [41,42].

## 3. Results

### 3.1. Toxicity of Lambda-Cyhalothrin to A. gossypii

As depicted in Table 1 and Figure S1, the LC_30_, LC_50_, and LC_90_ values for adult *A. gossypii* exposed to lambda-cyhalothrin for 48 h were 1.15, 4.29, and 105.94 mg/L, respectively. The corresponding 95% confidence intervals were 0.47–2.04, 2.52–6.26, and 63.02–236.87 mg/L, respectively.

### 3.2. Effect of a Sublethal Concentration of Lambda-Cyhalothrin on the Growth and Development of F_0_ Generation of A. gossypii

**Figure 1:**
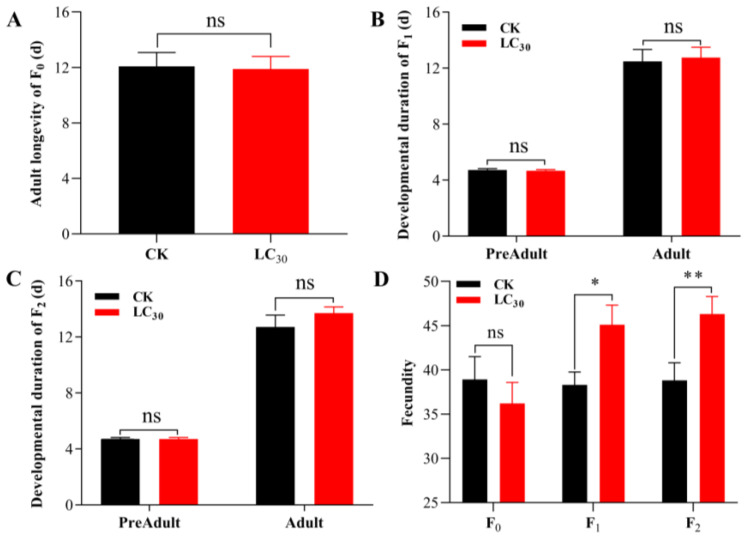
Developmental duration (**A**–**C**) and fecundity (**D**) of F_0_–F_2_ generations of *A. gossypii* treated with LC_30_ of lambda-cyhalothrin. Means ± SE in the same row followed by the same lowercase letters represent significant differences between treatments using a paired bootstrap test (ns denotes insignificance, * denotes *p*-value less than 0.05, and ** denotes *p*-value less than 0.01).

Adult longevity was not significantly different between the LC_30_ lambda-cyhalothrin treated control group and the F_0_ generation of *A. gossypii* (Figure 1A). And the fecundity was slightly lower in the treatment group, but the difference was not statistically significant (Figure 1D).

There was also a difference between the sublethal doses of lambda-cyhalothrin and control group in terms of *A. gossypii* generation F_0_ population growth parameters was also observed. After treatment, both the *r* and *λ* were markedly lower in the treatment group, and the *T* was significantly prolonged. However, the *R*_0_ difference is not significant (Table 2).

**Figure 2:**
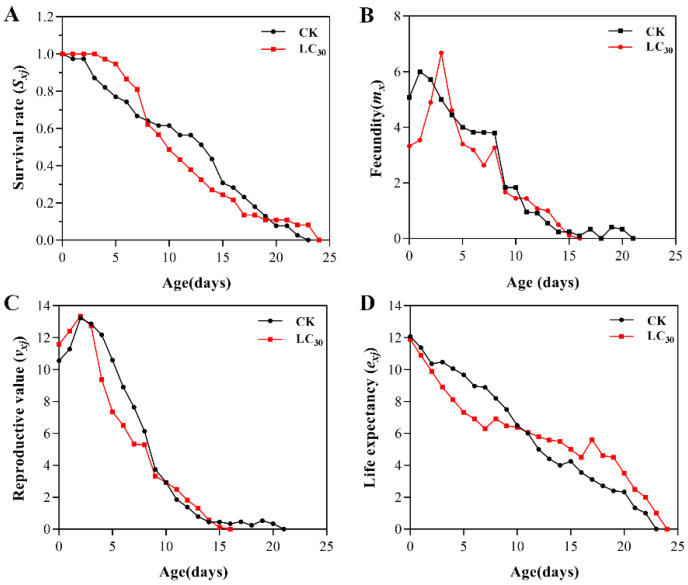
Age-stage specific survival rate (*S_xj_*) (**A**), age-specific fecundity (*m_x_*) (**B**), age-stage reproductive value (*v_xj_*) (**C**), and age-stage specific life expectancy (*e_xj_*) (**D**) of F_0_ generation of *A. gossypii* treated with LC_30_ of lambda-cyhalothrin.

The *S_xj_* curve of the F_0_ generation of *A. gossypii* in the treatment group exhibited a similar decreasing trend to the control (Figure 2A). This indicated that the survival rate of *A. gossypii* was minimally affected by the LC_30_ of lambda-cyhalothrin. The *m_x_* curves showed a rise at first, then a decline, reaching a peak at the age of 4 days (6.68 offspring) for the LC_30_ treatment and at the age of 3 days (6.00 offspring) in the control. Notably, LC_30_ treatment group had a higher peak fecundity than that of the control group (Figure 2B). The *v_xj_* curves exhibited an initial rise followed by a decline over time, with both the treatment and control groups reaching their peak of reproduction values at 3 days (13.36 and 13.23 offspring, respectively) (Figure 2C). Adult life expectancy was 11.89 days in the treatment group and 12.08 days in the control (Figure 2D).

**Figure 3:**
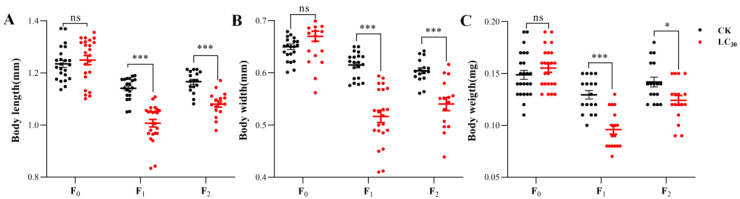
Body length (**A**), body width (**B**), and body weight (**C**) of F_0_–F_2_ generation of *A. gossypii* treated with sublethal concentration of lambda-cyhalothrin. The Student’s *t*-test is used for significance analysis (ns denotes insignificance, * denotes *p*-value less than 0.05, and *** denotes *p*-value less than 0.005). All error bars indicate ±SE.

There were no significant differences in body length, width, or weight between the treatment and control cohorts (Figure 3).

### 3.3. Effects of a Sublethal Concentration of Lambda-Cyhalothrin on the Growth and Development of F_1_ Generation of A. gossypii

*A. gossypii*’ s F_1_ generation development was not significantly different from that of the control group in LC_30_ lambda-cyhalothrin treatment (Figure 1B and Figure S2A). However, there was a significant difference between the treatment and control groups in terms of fecundity (Figure 1D).

Although the control group exhibited no significant differences in *r*, *λ*, and *T*, the *R*_0_ of the F_1_ generation of *A. gossypii* in the LC_30_ lambda-cyhalothrin treatment exhibited a significant increase (Table 3).

**Figure 4:**
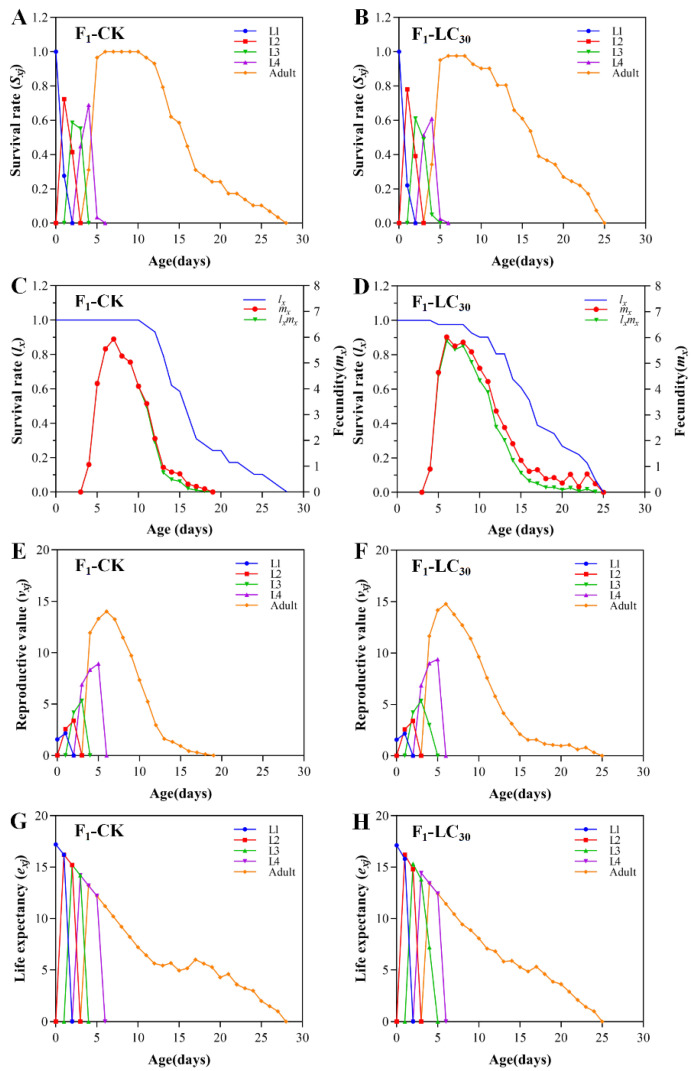
Age-stage specific survival rate (*S_xj_*) (**A**,**B**), age-specific survival rate (*l_x_*), age-specific fecundity (*m_x_*), age-specific maternity (*l_x_m_x_*) (**C**,**D**), age-stage reproductive value (*v_xj_*) (**E**,**F**), and age-stage specific life expectancy (*e_xj_*) (**G**,**H**) of F_1_ generation of *A. gossypii* treated with LC_30_ of lambda-cyhalothrin.

The *S_xj_* curves of F_1_ generation of *A. gossypii* in the treatment group overlapped with the *S_xj_* curves of the control group (Figure 4A,B). This suggested that the survival rate of the F_1_ generation in the treatment group was almost the same as that of the control group at any stage. Likewise, the *l_x_* curves of the experimental group almost overlapped with the control group. The *m_x_* curves showed a rise at first, then a decline, with the treatment group reaching its peak at 6 days old (6.02 offspring), and the control group showing a peak at 7 days old (5.93 offspring) (Figure 4C,D). The *v_xj_* curves (Figure 4E,F) showed a significant increase in reproductive values after reaching adulthood, followed by a decline, as the fecundity decreased. In both treatment and control groups, peak reproductive values were observed on the sixth day, with values of 14.78 and 14.01 offspring, respectively. Notably, the peaks and timing of peak occurrence were similar. The life expectancy of the treatment and control groups were 17.12 and 17.21 days, respectively (Figure 4G,H).

*A. gossypii* F_1_ generation body length, width, and weight were significantly lower than those of the control group in the treatment group (Figure 3).

### 3.4. Effects of a Sublethal Concentration of Lambda-Cyhalothrin on the Growth and Development of F_2_ Generation of A. gossypii

The development of each stage of the F_2_ generation of *A. gossypii* in the treatment group was similar to that in the control (Figure 1C and Figure S2B), whereas fecundity was significantly higher (Figure 1D).

The population growth parameters of F_2_ generation of *A. gossypii* are shown in Table 3. The *R*_0_, *r*, and *λ* in the LC_30_-treated F_2_ generation of *A. gossypii* were not significantly different from those of the control, but the *T* was markedly prolonged.

**Figure 5:**
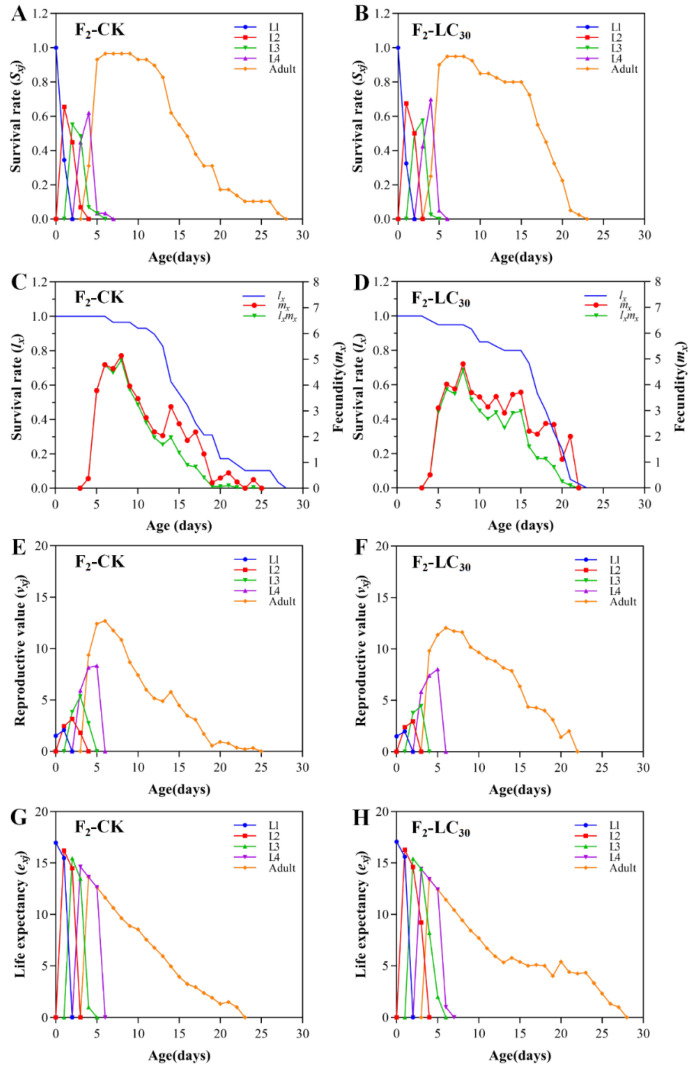
Age-stage specific survival rate (*S_x_*_j_) (**A**,**B**), age-specific survival rate (*l_x_*), age-specific fecundity (*m_x_*), age-specific maternity (*l_x_m_x_*) (**C**,**D**), age-stage reproductive value (*v_xj_*) (**E**,**F**), and age-stage specific life expectancy (*e_xj_*) (**G**,**H**) of F_2_ generation of *A. gossypii* treated with LC_30_ of lambda-cyhalothrin.

The *S_xj_* curve of the F_2_ generation of *A. gossypii* treated with LC_30_ lambda-cyhalothrin almost overlapped with that of the control group (Figure 5A,B). This indicates that the survival rate of the treatment group at each stage is the same as that of the control group. Additionally, the comparison between the control group and the treatment group showed similar *l_x_* curves, whereas the *m_x_* curves initially increased and then decreased. The treatment group and the control group both reached their peak at 8 days of age (4.85 and 5.14 offspring, respectively) (Figure 5C,D). Furthermore, the *v_xj_* curves indicated a substantial rise in the reproductive values of both the treatment and control groups upon reaching adulthood, followed by a subsequent decline coinciding with a decrease in fecundity. The groups reached their peak reproductive values at the age of 7 days (11.56 offspring) and 6 days (12.05 offspring), respectively (Figure 5E,F). Additionally, the life expectancy of the treatment and control groups were 17.72 and 17.07 days, respectively (Figure 5G,H).

The body length, width, and weight of the F_2_ generation of *A. gossypii* in the LC_30_ lambda-cyhalothrin treatment were significantly reduced compared to those of the control (Figure 3).

### 3.5. Effect of a Sublethal Concentration of Lambda-Cyhalothrin on the Expression Level of A. gossypii Vg and VgR

**Figure 6:**
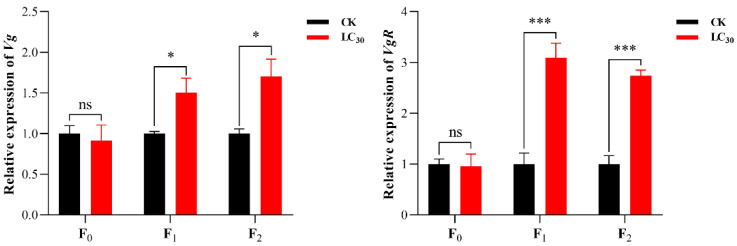
Relative expression level of *Vg* (**A**) and *VgR* (**B**) in F_0_–F_2_ generations of *A. gossypii.* The Student’s *t*-test is used for significance analysis (ns denotes insignificance, * denotes *p*-value less than 0.05, and *** denotes *p*-value less than 0.005). All error bars indicate ±SE.

*Vg* and *VgR* gene expression in F_0_–F_2_ generations of *A. gossypii* treated with LC_30_ lambda-cyhalothrin is shown in Figure 6. A significant difference was not found between the treatment and control groups in the relative expression levels of the *Vg* and *VgR* genes, whereas *Vg* and *VgR* gene expression was significantly elevated in the F_1_ and F_2_ generations treatment groups compared with that of controls.

## 4. Discussion

Exposure to low doses of insecticides induces varying degrees of sublethal effects, which may result in a reduction or increase in pest offspring [43]. This phenomenon has been reported in studies involving Aphididae after treatment with insecticides such as azinphos methyl [44], azadirachtin [45], imidacloprid [46], thiamethoxam [47], nitenpyram [6], flupyradifurone [48,49], sulfoxaflor [29,50], and decamethrin [9].

In this study, the treatment of the F_0_ generation of *A. gossypii* with LC_30_ of lambda-cyhalothrin resulted in a decrease in fecundity and *R*_0_, although these differences were statistically insignificant compared to the control. Additionally, a significant reduction in *r* and *λ* and a pronounced prolongation in *T* were observed. It is possible that *A. gossypii* adapted to the stress of lambda-cyhalothrin to improve survival by reducing the reproductive ability, indicating that lambda-cyhalothrin at LC_30_ can inhibit the reproduction of *A. gossypii* in the F_0_ generation, aligning with the research of Wang et al. [6]. However, flupyradifurone at concentrations LC_10_ and LC_25_ significantly increased the adult longevity and fecundity of *A. gossypii* [49], which could be attributed to the varying sublethal impacts of distinct insecticides on *A. gossypii*.

In this study, the F_1_ and F_2_ generations of *A. gossypii* showed a reduction in body length, width, and weight, as well as a significant increase in fecundity, after treatment with LC_30_ of lambda-cyhalothrin, indicating that the sublethal concentration of lambda-cyhalothrin had a positive impact on their reproductive capacity. Similarly, sulfoxaflor [50] at LC_20_, decamethrin at LC_30_, and imidacloprid [51] at LC_30_ increased the fecundity of F_1_ *Myzus persicae*, whereas imidacloprid [52] and acetamiprid [53] at LC_15_ increased the fecundity of F_1_ *A. gossypii*. This could be due to a disruption in physiological equilibrium caused by insecticide stress in the F_0_ generation, which inhibited F_0_ reproduction. Additionally, the embryos in the abdomen of the F_0_ generation of *A. gossypii* absorbed a small amount of lambda-cyhalothrin from their mothers [6]. The F_1_ generation then increased its reproduction rate through the overcompensation effect, and the body length, width, and weight were significantly reduced. Moreover, with the superposition of generations, this effect continued throughout the F_2_ generation. This aligns with the discoveries made by Shang et al. [9]. In contrast, treating *A. gossypii* with buprofezin significantly reduced the *R*_0_, *r*, *λ*, and total fecundity, as well as significantly increased the mean generation time in the F_1_ generation [54]. It is hypothesized that differences in chemical insecticide types, exposure modes, and exposure times led to some variation in the outcomes of the aforementioned studies.

The *Vg* and *VgR* are frequently used to assess the fecundity of female insects. The expression levels of the insect *Vg* and *VgR* genes have been shown to be affected by sublethal doses of insecticides. The RT-qPCR analysis in this study revealed that *Vg* and *VgR* gene expression levels were not significantly different in the F_0_ generation of *A. gossypii*. F_1_ and F_2_ generation *A. gossypii* showed significantly increased expression of the *Vg* and *VgR* genes, in line with the results of studies on *A. gossypii* treated with the sublethal concentrations of acetamiprids [53]. Therefore, lambda-cyhalothrin did not induce the expression of the *Vg* and *VgR* genes in the F_0_ generation of *A. gossypii*. However, the expression of *Vg* and *VgR* genes in F_1_ and F_2_ generations of *A. gossypii* can be stimulated in alternate generations, which improves their fecundity and allows them to adapt to lambda-cyhalothrin stress.

In summary, the offspring of *A. gossypii* exposed to sublethal doses of lambda-cyhalothrin will re-emerge. On the one hand, lambda-cyhalothrin has a stimulating effect on the reproduction of *A. gossypii*, and on the other hand, that lambda-cyhalothrin may develop resistance to *A. gossypii*. Therefore, it is difficult to control *A. gossypii*. We speculate that this should also be one of the reasons for the current decrease in the amount of pyrethroid pesticides. This study provides a theoretical basis for the rational use of pesticides in the field.

## 5. Conclusions

In this study, the sublethal and intergenerational effects of lambda-cyhalothrin exposure on *A. gossypii* were investigated using a life table and qPCR. The results showed that the sublethal doses of lambda-cyhalothrin inhibited the reproduction of the parental generation of *A. gossypii* but enhanced the reproductive potential of the subsequent generations (F_1_ and F_2_). Furthermore, the changes in *Vg* and *VgR* of *A. gossypii* may be closely related to its sublethal effects. Therefore, the purpose of this study is to provide a theoretical foundation for the effective use of pesticides in the control of *A. gossypii*, as well as for the development of strategies to manage resistance to pesticides.

## Figures and Tables

**Table 1:** Toxicity of lambda-cyhalothrin to *A. gossypii.*

N ^a^	Slope ± SE ^b^	LC_30_ (mg/L) (95%CL) ^c^	LC_50_ (mg/L) (95%CL)	LC_90_ (mg/L) (95%CL)	χ^2 d^	R^2^
840	0.92 ± 0.12	1.15 (0.47–2.04)	4.29 (2.52–6.26)	105.94 (63.02–236.87)	1.48	0.98

Note: ^a^ number of *A. gossypii* adults; ^b^ standard error; ^c^ 95% confidence limits; and ^d^ Chi-square value (χ^2^).

**Table 2:** Population growth parameters of F_0_ generation of *A. gossypii* exposed to LC_30_ of lambda-cyhalothrin.

Parameters	CK		LC_30_		*p*
	N	Mean ± SE	N	Mean ± SE	
*R*_0_	78	38.92 ± 2.58	74	36.21 ± 2.38	0.44
*r*	78	0.4303 ± 0.0054	74	0.4082 ± 0.0040	0.01
*λ*	78	1.5377 ± 0.0082	74	1.5040 ± 0.0061	0.01
*T*	78	8.50 ± 0.10	74	8.79 ± 0.10	0.05

Note: *R*_0_, net reproductive rate (offspring/female); *r*, intrinsic rate of increase (d^−1^); *λ*, finite rate of increase (d^−1^); and *T*, mean generation time (d). The table displays the mean ± SE.

**Table 3:** Population growth parameters of F_1_–F_2_ generation of *A. gossypii* treated with sublethal concentration of lambda-cyhalothrin.

Parameters	F_1_					F_2_				
	CK		LC_30_		*p*	CK		LC_30_		*p*
	N	Mean ± SE	N	Mean ± SE		N	Mean ± SE	N	Mean ± SE	
*R*_0_	87	38.31 ± 1.45	82	44.00 ± 2.44	0.04	87	37.48 ± 2.33	80	43.73 ± 2.71	0.08
*r*	87	0.4492 ± 0.0072	82	0.4492 ± 0.0072	0.76	87	0.4198 ± 0.0089	80	0.4050 ± 0.0119	0.32
*λ*	87	1.5672 ± 0.0113	82	1.5724 ± 0.0123	0.76	87	1.5216 ± 0.0135	80	1.4995 ± 0.0178	0.32
*T*	87	8.12 ± 0.13	82	8.36 ± 0.12	0.17	87	8.63 ± 0.17	80	9.33 ± 0.18	0.01

Note: *R*_0_, net reproductive rate (offspring/female); *r*, intrinsic rate of increase (d^−1^); *λ*, finite rate of increase (d^−1^); and *T*, mean generation time (d). The table displays the means ± SE.

## Data Availability

The datasets used and analyzed during the current study can be supplied by the corresponding author upon reasonable request.

## Supplementary Materials

The following supporting information can be downloaded at: https://www.mdpi.com/article/10.3390/insects15030173/s1, Figure S1: Regression line indicating the relationship between the log of concentrations of lambda-cyhalothrin and the mortality rate of *A. gossypii* after 48 h. Figure S2: The developmental duration of nymphs of F_1_ and F_2_ generations of *A. gossypii* treated with the sublethal concentration of lambda-cyhalothrin. Means ± SE in the same row followed by the same lowercase letters represent significant differences between treatments using a paired bootstrap test. ns indicates not significant. Table S1: Primers used for the RT-qPCR.
