# Supplementary material for: Intergenerational Effects of Sublethal Lambda-Cyhalothrin Exposure on Aphis gossypii Glover (Hemiptera: Aphididae) Reproduction and Development
Source: Insects. 2024 Mar 4;15(3):173. doi: 10.3390/insects15030173 (PMC10971360; doi:10.3390/insects15030173)
Supplement: Supplementary file 1 [file insects-15-00173-s001.zip › insects-2883536-supplementary.pdf]

Supplementary Information

Intergenerational Effects of Sublethal Lambda-Cyhalothrin Exposure on *Aphis gossypii* Glover (Hemiptera: Aphididae) Reproduction and Development

Yuepeng Qiu * and Zhaorong Chen *

(College of Horticulture and Landscape, Tianjin Agricultural University, Tianjin 300392, China)

This Supplementary Information contains the following:
Table S1. Primers used for the RT-qPCR.
Figure S1. Regression line indicating the relationship between the log of concentrations of lambda-cyhalothrin and the mortality rate of *A. gossypii* after 48 h.
Figure S2. The developmental duration of nymphs of F_1 and F_2 generations of *A. gossypii* treated with the sublethal concentration of lambda-cyhalothrin.

Table S1. Primers used for the RT-qPCR.

Primer name	Primer sequence
Vg-F	5’-ACCACTGCACACTCGGATAA-3’
Vg-R	5’-CGGCTTGCATGAACCAGTAG-3’
VgR-F	5’-CAGGGCGAACGTCCATACAAC-3’
VgR-R	5’-GTATCCACTGCAATCAGATTGC-3’
β-actin-F	5’-TGGACTCTGGTGACGGTGTCTC-3’
β-actin-R	5’-ATTTCTCTTTCAGCGGTGGTGG-3’

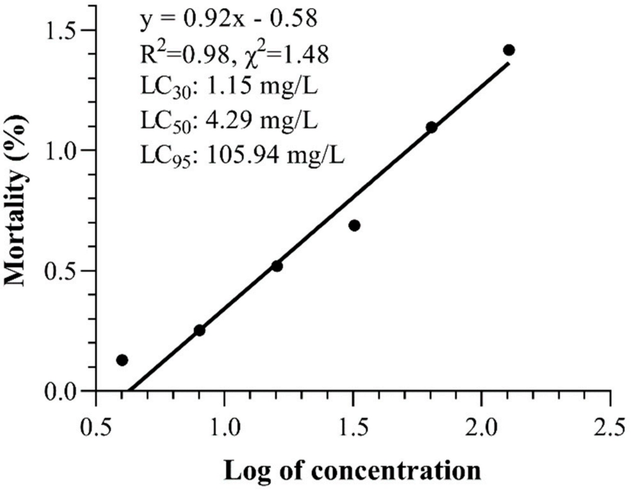

Figure S1. Regression line indicating the relationship between the log of concentrations of lambda-cyhalothrin and the mortality rate of *A. gossypii* after 48 h.

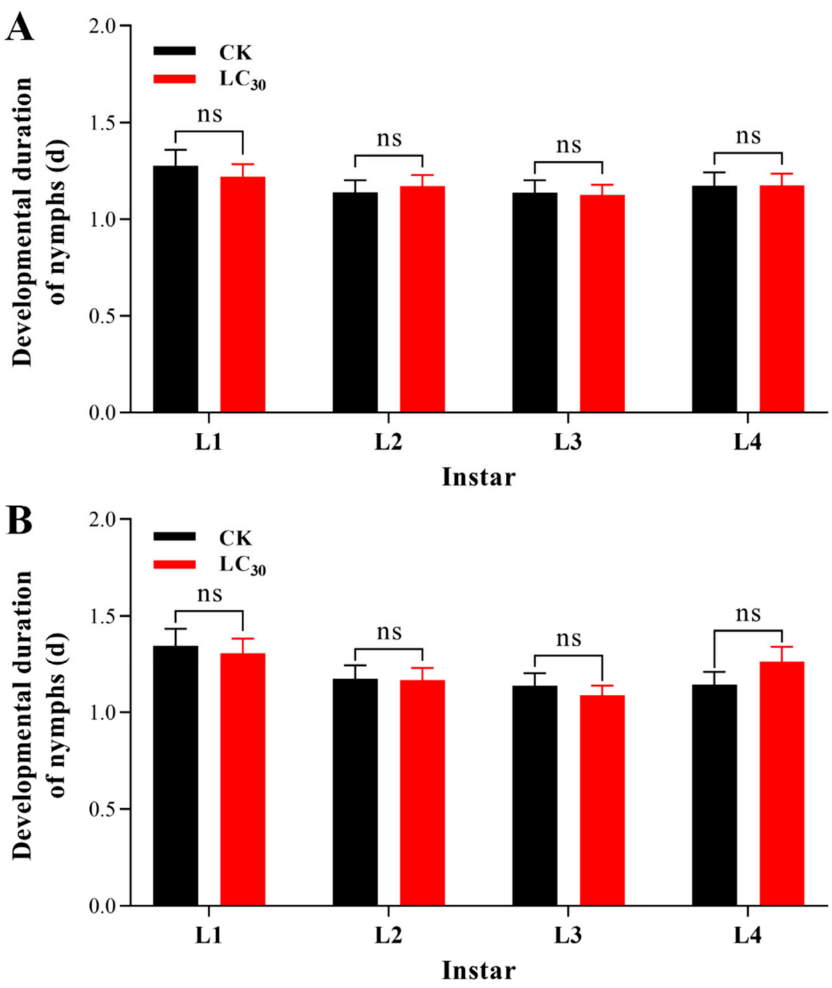

Figure S2. The developmental duration of nymphs of F_1 (**A**) and F_2 (**B**) generations of *A. gossypii* treated with the sublethal concentration of lambda-cyhalothrin. Means ± SE in the same row followed by the same lowercase letters represent significant differences between treatments using a paired bootstrap test. ns indicates not significant.
